# Tumor cell expression of CD163 is associated to postoperative radiotherapy and poor prognosis in patients with breast cancer treated with breast-conserving surgery

**DOI:** 10.1007/s00432-018-2646-0

**Published:** 2018-05-03

**Authors:** Stina Garvin, Husam Oda, Lars-Gunnar Arnesson, Annelie Lindström, Ivan Shabo

**Affiliations:** 1 Divison of Neurobiology, Department of Clinical and Experimental Medicine, Faculty of Health Sciences, Linköping University, Linköping, Sweden; 2 Department of Clinical Pathology, Centre for Diagnostics, Region Östergötland, Linköping, Sweden; 3 Department of Pathology, County Hospital Ryhov, Jönköping, Sweden; 4 Departement of Medical Biosciences, Pathology, Umeå University, Umeå, Sweden; 5 Division of Surgery, Department of Clinical and Experimental Medicine, Faculty of Health Sciences, Linköping University, Linköping, Sweden; 6 Division of Cell Biology, Department of Clinical and Experimental Medicine, Faculty of Health Sciences, Linköping University, Linköping, Sweden; 7 Endocrine and Sarcoma Surgery Unit, Department of Molecular Medicine and Surgery, Karolinska Institutet, 171 77 Stockholm, Sweden; 8 Department of Breast, Endocrine and Sarcoma Surgery, Karolinska University Hospital, Stockholm, Sweden

**Keywords:** Tumor-associated macrophages, CD163, Breast cancer, Radiotherapy, Treatment resistance

## Abstract

**Purpose:**

Cancer cell fusion with macrophages results in highly tumorigenic hybrids that acquire genetic and phenotypic characteristics from both maternal cells. Macrophage traits, exemplified by CD163 expression, in tumor cells are associated with advanced stages and poor prognosis in breast cancer (BC). In vitro data suggest that cancer cells expressing CD163 acquire radioresistance.

**Methods:**

Tissue microarray was constructed from primary BC obtained from 83 patients treated with breast-conserving surgery, 50% having received postoperative radiotherapy (RT) and none of the patients had lymph node or distant metastasis. Immunostaining of CD163 in cancer cells and macrophage infiltration (MI) in tumor stroma were evaluated. Macrophage:MCF-7 hybrids were generated by spontaneous in vitro cell fusion. After irradiation (0, 2.5 and 5 Gy γ-radiation), both hybrids and their maternal MCF-7 cells were examined by clonogenic survival.

**Results:**

CD163-expression by cancer cells was significantly associated with MI and clinicopathological data. Patients with CD163-positive tumors had significantly shorter disease-free survival (DFS) after RT. In vitro generated macrophage:MCF-7 hybrids developed radioresistance and exhibited better survival and colony forming ability after radiation compared to maternal MCF-7 cancer cells.

**Conclusions:**

Our results suggest that macrophage phenotype in tumor cells results in radioresistance in breast cancer and shorter DFS after radiotherapy.

## Introduction

Despite advances in early diagnosis and treatment of breast cancer (BC), about 15% of patients with localized breast cancer develop recurrent disease within 2–5 years of completed treatment (Pan et al. 2017; Touboul et al. 1999). The rates of local and systemic BC recurrence vary in different studies, but distant recurrence is the most common, illustrating that BC is often a systemic disease (Elsayed et al. 2016; Mamounas et al. 2014). Postoperative radiotherapy (RT) is an important complement to breast-conserving surgery. The purpose of RT is to kill cancer cells by inducing DNA-damage and eliminate microscopic tumor foci in the conserved breast (Clarke et al. 2005; Maier et al. 2016). In later stages of disease, the selection of therapy-resistant cell clones is thought to contribute to tumor recurrence and metastasis (Gonzalez-Angulo et al. 2007; Vrieling et al. 2003).

Tumor-associated macrophages (TAMs) are an important component of solid tumors (Komohara et al. 2016). Their presence in tumor stroma has been shown to be correlated with advanced tumor stages and progression in colorectal cancer and breast cancer (Leek et al. 1996; Shabo et al. 2014). Better understanding of the interaction between non-malignant inflammatory cells and tumor cells has yielded great progress in the field of immunotherapy in recent years (Golan et al. 2017). Tumor-stroma cell fusion has been proposed as a potential mechanism to generates hybrids with genetic and phenotypic characteristics from both maternal cells (Busund et al. 2002; Powell et al. 2011; Shabo et al. 2015). Macrophage phenotype in cancer cells, detected by CD163-expression, is suggested to be caused by fusion between TAMs and cancer cells (Powell et al. 2011; Shabo et al. 2015). In vitro and in vivo experimental data supports that cell fusion occurs in solid tumors and may play a significant role in clinical tumor progression (Powell et al. 2011). Moreover, cancer cell fusion has been shown to contribute to tumor heterogeneity, creating subsets of tumor cells with reduced susceptibility to hormone- and chemotherapy (Kaur et al. 2015; Lindstrom et al. 2017; Wang et al. 2012; Yang et al. 2010).

The aim of this study was to investigate the associations between MI, macrophage traits of breast cancer cells (as defined by CD163-expression), clinicopathological data, and disease recurrence in relation to RT in a well-defined patient cohort treated with breast-conserving surgery for non-metastatic breast cancer. Using this retrospective design, we were able to include patients who were not offered postoperative radiotherapy, as it was not fully implemented in clinical routine until the early 1990s, thus allowing for investigations into possible associations between CD163-expression/MI and recurrence in relation to radiotherapy (Fredriksson et al. 2001). To further explore the hypothesis of cell fusion between macrophages and cancer cells as an underlying mechanism of poor radiation response in the patient with CD163-positive tumors, an in vitro study was designed using GFP as a marker of maternal MCF-7 cells and CD163 as macrophage maker. Macrophage:MCF-7 hybrids (GFP- and CD163-positive) were collected and their radiosensitivity investigated in relation to maternal MCF-7 cells.

## Materials and methods

### Patient material and study design

We collected data on all patients (*n* = 1164) with BC with isolated ipsilateral local recurrence (ILR) during the years of 1983–2008 from the breast cancer registry of the southeastern region of Sweden. For comparison, we selected an age-matched patient cohort (*n* = 1164), treated during the same period and without ILR. Only patients with radically removed tumors (R0), without lymph node metastases (N0) or distant metastases (M0) were included. All patients were treated with conventional breast-conserving surgery at surgical departments within the county of Östergötland, Sweden. Ethical approval from the Regional Ethics Committee in Linköping was obtained according to Swedish Biobank Law (Reference Number 2010/311–31). All data are presented in the main manuscript.

Tumor histology was reviewed by an experienced pathologist (SG), and formalin-fixed paraffin-embedded tissue blocks with invasive BC were chosen for tissue microarray, constructed using two tissue cores (diameter 0.6 mm). Eighty-three patient samples were included in total. Liver samples were used as a position control.

### Immunostaining and evaluation

CD163 is considered as a macrophage-specific marker and is generally not expressed in cell types other than monocytes/macrophages. Based on the cell fusion theory, we used CD163-expression as a surrogate marker for macrophage phenotype in breast cancer cells. Five micrometer sections were obtained from formalin-fixed paraffin-embedded TMA tumor specimens. The sections were de-paraffinized in xylene and hydrated in a series of graded alcohols, pretreated with heat induced epitope retrieval and Tris-ethylenediamine tetraacetate acid buffer (1 mM, pH 9, 20/5/20 min; Decloaking Chamber NxGen, Biocare Medical), and stained for CD163 (anti-human, monoclonal antibody, clone 10D6, Novocastra, Leica). Staining for estrogen receptor (ER; clone SP1, Ventana Roche), progesterone receptor (PR; clone 1E2, Ventana Roche), Ki-67 (clone MIB-1, Dako Agilent), and human epidermal growth factor receptor 2 (HER2; clone 4B5, Ventana Roche) was done according to clinical laboratory standards. All slides were scanned to digital images using the Hamamtsu NanoZoomer XL (Visiopharm LRI AB). Image analysis and evaluation of immunostaining were performed by ImageScope viewing software (Leica Biosystems).

**Fig. 1:**
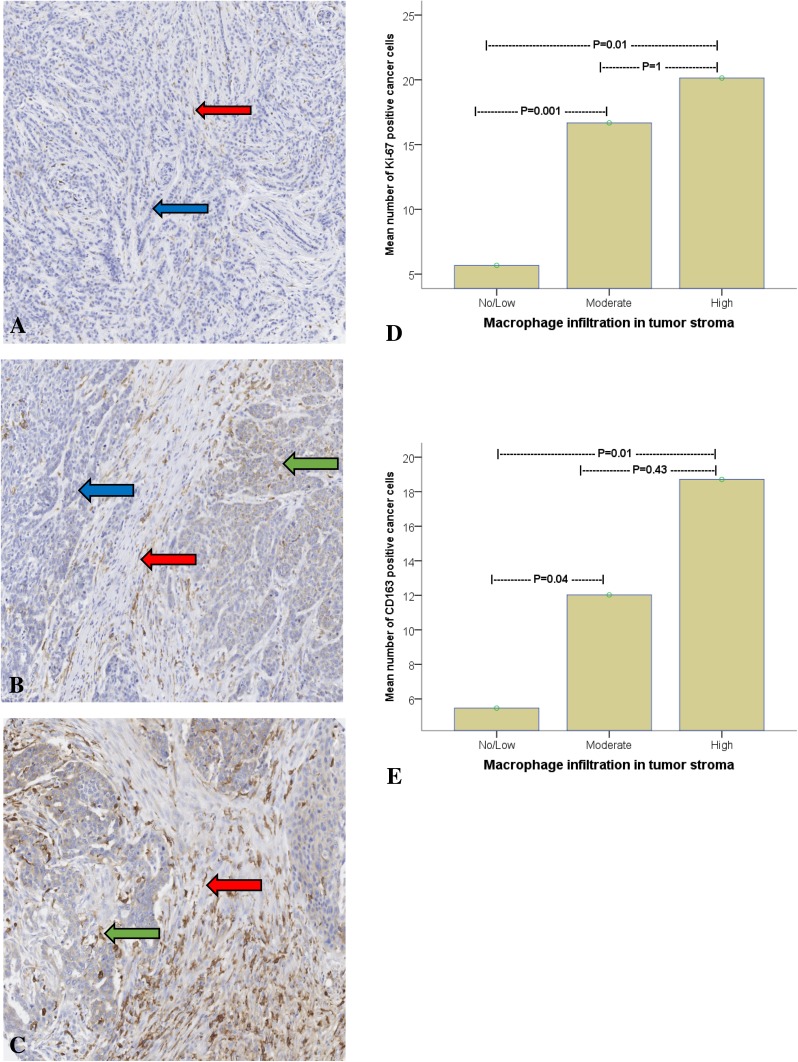
Infiltration of tumor-associated CD163-macrophages in breast cancer graded as no/low (**a**), moderate (**b**), or high (**c**); macrophages are indicated by red arrow. 17 of the 81 patients had CD163-positive tumors; example of CD163-positive cancer cell indicated by green arrow. The blue arrow shows a CD163-negative tumour cell. Analysis of variance (ANOVA) evaluating the association between macrophage infiltration and Ki-67 expression (**d**) and breast cancer cell CD163-expression (**e**)

All immunostaining was evaluated by two experienced pathologists (SG and HO), blinded to patient characteristics and outcome. Macrophages and cancer cells were distinguished histomorphologically, the macrophages exhibiting small, regular nuclei and the cancer cells atypical nuclei with variations in size, shape, and chromatin staining. TAM-infiltration was evaluated semi-quantitatively, classified in three grades: no/low, moderate, or high (Fig. 1a–c). The fraction of CD163-positive cancer cells was calculated based on a count of 200 tumor cells in each TMA core. The tumors were considered CD163-positive if > 15% of the tumor cells expressed CD163. The expression of Ki-67, ER, PR, and HER-2 in cancer cells was evaluated according to ESMO guidelines (2015) (Senkus et al. 2015).

### Cell line and monocyte isolation

MCF-7/green fluorescent protein (GFP)-breast cancer cell line (AKR-211, Cell Biolabs, Inc., USA) was cultured in Roswell Park Memorial Institute (RPMI) 1640 medium supplemented with 1% penicillin–streptomycin (PEST) (Thermo Fisher Scientific, USA), 10% Fetal Bovine Serum (FBS), and GlutaMax (Gibco^®^, Life Technologies, USA) in T-75 tissue culture flasks (Sigma–Aldrich, USA) and incubated at 37 °C 5% CO_2_ atmosphere. Cell medium was changed every 2–3 days, and the cells were passaged with 0.25% trypsin (Gibco, USA) at 95% confluence.

Monocytes were isolated from buffy coat obtained from healthy male blood donors at the department of transfusion medicine at Linköping University Hospital (Linköping, Sweden) and county hospital Ryhov (Jönköping, Sweden). All the blood donors gave informed consent according to the local guidelines and the Swedish National Law on ethical review of research involving humans (2003:460: 3–4§). The buffy coat was mixed with 70 ml 0.9% NaCl, layered onto 20 ml Lymphoprep (Thermo Fisher Scientific, USA) in 50 ml centrifuge tubes, and centrifuged at 480*g* in room temperature for 40 min. The buffy coat layer was transferred into new 50 ml tubes containing PBS-Heparin [500 ml PBS, pH 7.3, and 50 µl Heparin (0.01% Heparin 5000 IE/ml; Medicago Leo Pharma, Denmark)] and centrifuged at 300*g* for 10 min at 4 °C. The cell pellets were washed twice in PBS-Heparin (220 g, 5 min, 4 °C), followed by three washing procedures in Krebs–Ringer bicarbonate buffer (Sigma–Aldrich, USA) without Ca^2+^ (220 g, 5 min, 4 °C). White blood cells were re-suspended in 20 ml RPMI1640 medium supplemented with 1% PEST, seeded into T-75 tissue culture flasks, and incubated for 1–2 h at 37 °C with 5% CO_2_ to allow monocyte adhesion. The non-adherent cells were eliminated by washing 2–3 times using PBS 37 °C and remaining attached cells incubated for 24 h at 37 °C with 5% CO_2_ before differentiation to macrophages by incubation (at 37 °C in 5% CO_2_) with 40 ng/ml of macrophage colony-stimulating factor, M-CSF (Nordic Biosite, Sweden), for 5–7 days and thereafter induced to M2 polarization with 20 ng/ml human interleukin-4 (Nordic Biosite, Sweden) for 18–24 h.

### Macrophage/MCF-7 fusion

Spontaneous cell fusion occurred between macrophages and MCF-7/GFP-cancer cells upon co-culturing the cells at a ratio 3–5:1 (macrophage:MCF-7) in RPMI 1640 medium (supplemented with 10% FBS, 5% PEST, GlutaMax) at 37 °C for 2 days. The cells were harvested with a 0.05% trypsin–EDTA solution (Gibco, USA), centrifuged at 300*g* for 5 min at 4 °C, washed with 1 ml PBS 4 °C, and resuspended in 95 µl cell staining buffer (Nordic Biosite, Sweden) at a concentration of approximately 5 × 10^6^ cells/ml. The cell suspension was incubated on ice for 10 min with 5 µl TrueStainFcX solution (BioLegend, USA). Combinations of direct conjugated monoclonal anti-human CD163 (APC Anti-human CD163 (IgG1 k), clone GHI/61, 100 µg/ml) and anti-human CD45 (CF405M anti-human CD45 (IgG1 k), clone HI30, 50 µg/ml) antibodies or their respective isotype controls (APC and CF405M mouse IgG1 k, clone MOPC-21, 200 µg/ml; all antibodies from Biolegend, USA) were added to the cell suspension at concentrations recommended by the manufacturer and incubated at 4 °C for 30 min in darkness. The samples were centrifuged at 300*g* for 5 min at 4 °C and excess of antibodies was removed. The labelled cells were washed twice in 1 ml cell staining buffer, diluted in 1 ml PBS, and filtered in a pre-separation filter (30 µm, Miltenyi Biotech, Sweden) before they were sorted with BD FACSAria^™^ III (BD Bioscience, USA; violet laser 405 nm, blue laser 488 nm, green laser 561 nm, red laser 632 nm). The cells were initially sorted by GFP-expression (positive selection of MCF-7/GFP origin) and subsequently by CD163-and CD45-expression. Macrophage/MCF-7-hybrids were defined as expressing both GFP and macrophage markers (CD163 and CD45). Cells positive for these markers were collected in tubes (BD FalconTM, Thermo Fisher Scientific) containing 0.5 ml FBS at 4 °C.

### Radiation of cells and analysis of clonogenic survival

MCF-7/GFP-cells and M2-macrophage/MCF-7-hybrids (5 × 10^5^cells) were seeded in T-25 tissue culture flasks with RPMI 1640 medium and allowed to grow for 2 days (90–95% confluency). At day 3, the cell cultures were exposed to γ-radiation (Clinac 600C/D, Varian Medical Systems Incorporated, Herlev, Denmark, one AP field, linear accelerated 6MV Photons), at a dose-rate of 5 Gy/min and doses of 0 (control), 2.5 and 5.0 Gy at room temperature. The culture flasks were surrounded with 3 cm poly methyl methacrylate (PMMA) with a density comparable to that of human tissue.

After radiation procedure and storage at 4 °C, the cells were trypsinated and resuspended in RPMI medium. Cell counts were determined from two aliquots (TC10^™^ Automated Cell Counter, Bio-Rad Laboratories AB, Sweden). Mean was used to prepare triplicates of100 cells per each 60 mm petri dishes (150288 Nunc^™^, ThermoFischer Scientific, Denmark). The cultures were incubated with 4 ml RPMI medium (10% FBS, 5% PEST, GlutaMax) at 37 °C with 5% CO_2_ for 6 days. After incubation, the cultures were washed with PBS (Medicago, Sweden) followed by incubation for 30 min in 6% glutaraldehyde (Fisher Scientific GTF) and 0.5% Crystal Violet staining solution (ServaElectrophoresis GmbH, Germany). The dishes were washed with water and allowed to dry at room temperature in darkness. Colonies (> 50 cells/colony) were counted using a visible light source (Olympus CH-2, Japan). Plating efficiency (PE) was defined as the proportion of colonies developed from the seeded cells and calculated according to the equation: PE = number of colonies/number of seeded cells. The survival fraction (SF) was estimated as SF = number of colonies formed after irradiation/(number of seeded cells × PE/100)(Franken et al. 2006).

### Statistical analysis

SPSS statistics software, version 24(IBM Corporation, USA), was used for the statistical analyses. CD163-expression and MI were evaluated in relation to clinicopathologic data (in proportions) using Pearson’s Chi-square test. For continuous data, one-way analysis of variance (ANOVA) was used together with a post-hoc Bonferroni’s test. Survival rates were estimated according to Kaplan–Meier based on recurrence-free survival (RFS) and disease-free survival (DFS). The statistical significance of differences between survival rates was determined by the log-rank test. For all analyses, *p* < 0.05 (double-sided) was considered statistically significant.

## Results

### CD163-expression in breast cancer cells

CD163-expression in breast cancer cells was found in 19 (23%) of the patients. The mean proportion of CD163-positive cells in all tumors was 9% (range 0–41%). Two cases (2.4%), could not be evaluated for CD163-expression due to technical failure.CD163-expression > 15% was significantly associated with breast cancer-related death (*p* = 0.02). CD163-expression ≤ 15% correlated neither with breast cancer-related death nor other clinicopathological data. Thus, 15% was chosen as the cut-off for defining CD163-positivity in further analyses. Using this definition, 17 of the 81 patients (21%) had CD163-positive tumors (Table 1). CD163-expression was more common in poorly differentiated tumors. All 20 NHG1-tumors were CD163-negative while 10 of 25 of NHG3-tumors were CD163-positive. Similarly, a lower proliferative index as measured by Ki-67 was more common in the CD163-negative group (*p* = 0.008). CD163-expression did not appear to be related to T-stage, ER, or PR-status (Table 2).

**Table 1:** Patient characteristics

Variables	*N* (%)
Age groups (years)	
≤ 40	15 (18)
41–50	18 (22)
51–60	17 (20)
61–70	15 (18)
≥ 70	18 (22)
Pathologic T-stage	
pT1a	4 (5)
pT1b	23 (28)
pT1c	43 (51.2)
pT2	13 (15.5)
Nottingham grade	
NHG 1	20 (24)
NHG 2	38 (46)
NHG 3	25 (30)
ER-status	
Negative	14 (21)
Positive	66 (79)
Missing data	3
PR-status	
Negative	23 (28)
Positive	58 (72)
Missing data	2
HER2-status	
Negative	73 (92)
Positive	6 (8)
Missing data	4
Ki-67-expression	
≤ 15%	43 (56)
> 15%	34 (44)
Missing data	5
Postoperative radiotherapy	
No	42 (51)
Yes	41 (49)
Local recurrence	
No	44 (53)
Yes	39 (47)
Tumor cell CD163-expression	
Negative (≤ 15%)	64 (79)
Positive (> 15%)	17 (21)
Missing data	2
Macrophage infiltration	
No/low	41 (49)
Moderate	28 (36)
High	12 (15)
Missing data	2

**Table 2:** Univariate analysis of CD163-expression in tumor cells and macrophage infiltration in relation to clinicopathologic data in breast cancer

	TumorCD163-expression			Macrophage infiltration			
	≤ 15%, *n* (%)	> 15%, *n* (%)	*p*	No/low, *n* (%)	Moderate, *n* (%)	High, *n* (%)	*p*
Age groups (years)							
≤ 40	10 (16)	5 (29)		7 (17)	6 (21)	2 (17)	
41–50	15 (23)	2 (12)		7 (17)	8 (29)	2 (17)	
51–60	14 (22)	2 (12)		9 (22)	6 (21)	1 (8)	
61–70	11 (27)	4 (23)		10 (24)	3 (11)	2 (17)	
≥ 70	14 (22)	4 (24)	0.5	8 (20)	5 (18)	5 (42)	0.6
Pathologic T-stage							
pT1a	4 (6)	0 (0)		2 (5)	0 (0)	2 (17)	
pT1b	18 (28)	5 (29)		14 (34)	6 (21)	3 (25)	
pT1c	33 (52)	8 (47)		21 (51)	16 (57)	4 (33)	
pT2	9 (14)	4(24)	0.6	4 (10)	6 (22)	3 (25)	0.2
Nottingham grade							
NHG 1	20 (31.3)	0 (0)		17 (41)	2 (7.2)	1 (8)	
NHG 2	29 (45.3)	7 (41)		20 (49)	13 (46.4)	3 (25)	
NHG 3	15 (23.4)	10 (59)	0.004	4 (10)	13 (46.4)	8 (67)	< 0.001
ER-status							
Negative	9 (14)	5 (29)		2 (5)	8 (29)	4 (36)	
Positive	53 (86)	12 (71)	0.15	38 (95)	20 (71)	7 (64)	0.009
PR-status							
Negative	16 (25)	7 (41)		7 (17)	11 (39)	5 (45)	
Positive	47 (75)	10 (59)	0.2	34 (83)	17 (61)	6 (55)	0.06
HER2-status							
Negative	59 (97)	13 (81)		40 (98)	21 (84)	11 (100)	
Positive	2 (3)	3 (19)	0.03	1 (2)	4 (16)	0 (0)	0.06
Ki-67 index							
≤ 15%	37 (64)	4 (23)		29 (78)	11 (39)	1 (10)	
> 15%	21 (36)	13 (77)	0.003	8 (22)	17 (61)	9 (90)	< 0.001
Local recurrence							
No	36 (56)	8 (47)		23 (56)	15 (54)	6 (50)	
Yes	28 (44)	9 (53)	0.5	18 (44)	13 (46)	6 (50)	0.9

### Macrophage infiltration

MI was classified as low in 41 tumors (49%), moderate in 28 (34%), and high in 12 (15%). MI was also associated with poor differentiation/high grade (*p* < 0.001) and higher Ki-67. The mean number of cancer cells expressing Ki-67 was significantly lower in tumors with low MI compared to those with moderate (*p* = 0.001) and high (*p* = 0.01) MI (Fig. 1d). Logically, the expression of CD163 in cancer cells was also proportional to MI (*p* = 0.01 between low MI and high; Fig. 1e). The expression of ER (*p* = 0.009) and PR (*p* = 0.06) appeared to be inversely related to MI.

### CD163-expression and MI in relation to RFS and DFS

**Fig. 2:**
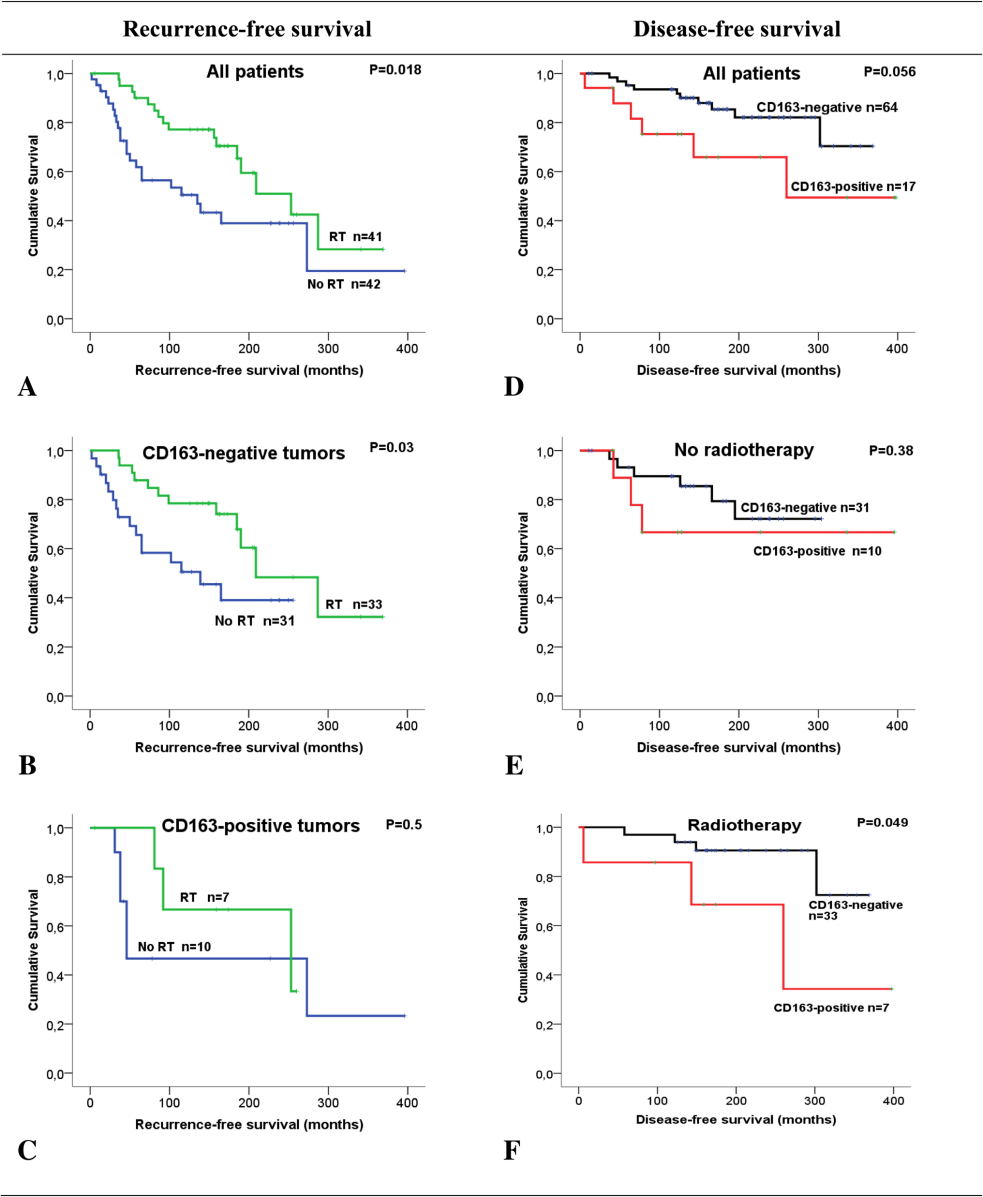
Survival analysis in breast cancer patients, treated with breast-conserving surgery, estimated as Kaplan Meier curves comparing ipsilateral local recurrence (**a**–**c**) and disease-free survival (**d**–**f**) in relation to postoperative radiotherapy and expression of CD163 in tumor cells

CD163-positivity was more common among those patients who experienced ILR (9/37, 24%) than among those who did not (8/44, 18%), but the difference was not statistically significant (*p* = 0.5; Table 2). As expected, RFS was significantly longer (231 months) in patients treated with RT compared to patients without RT (169 months, *p* = 0.018; Fig. 2). In patients with CD163-negative tumors, RFS was 140 months without RT and 237 months with RT (*p* = 0.03). The number of patients with CD163-positive tumors is relatively few, but the corresponding values in this group were 178 and 199 months, respectively (not significant).

Of the 17 patients with CD163-positive tumors, 6 (35%) patients died due to BC. Although not reaching statistical significance, the DFS appeared shorter in the group with CD163-positive tumors as compared with CD163-negative tumors (265 vs 316 months, *p* = 0.056; Fig. 2). No difference was found in the non-RT group, but in the group treated with postoperative radiotherapy, CD163-positivity was significantly associated with shorter DFS (251 vs 333 months, *p* = 0.049).

**Fig. 3:**
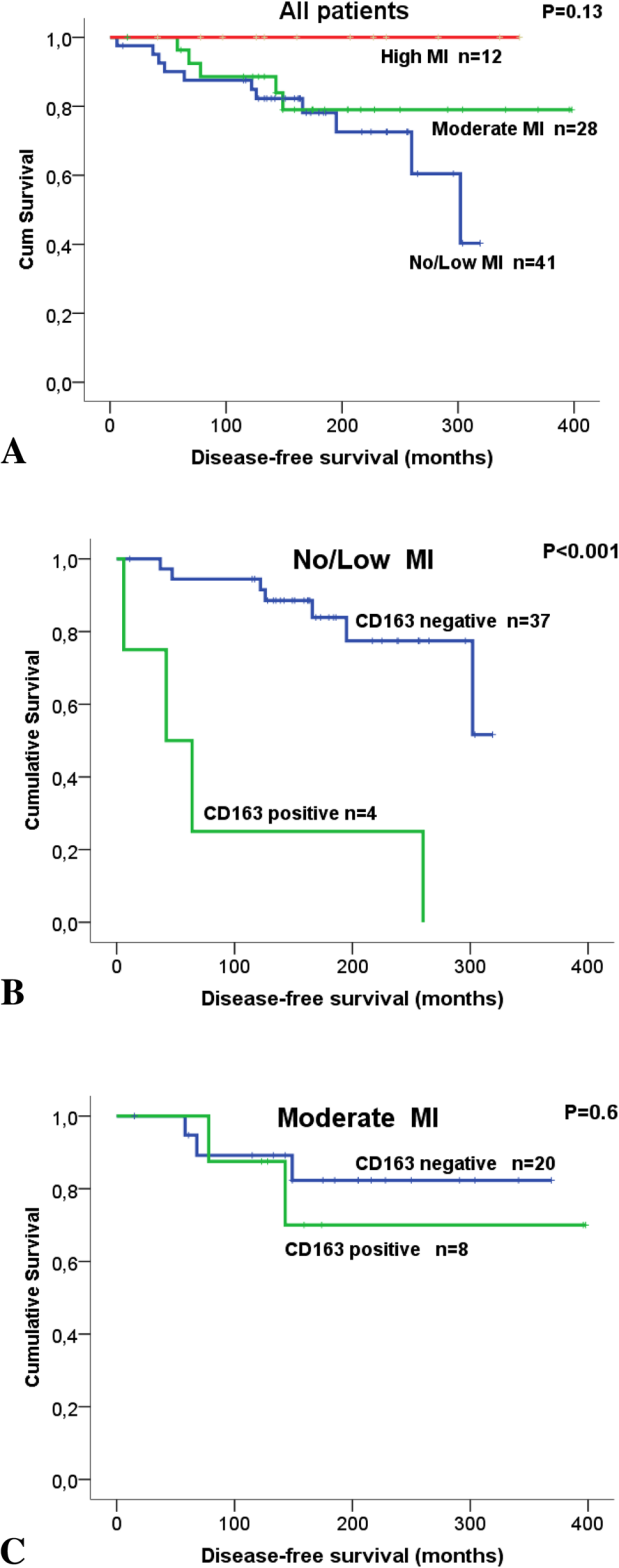
Survival analysis in breast cancer (BC) patients, treated with breast-conserving surgery, estimated as Kaplan Meier curves comparing disease-free survival (DFS) in relation to macrophage infiltration (MI) in tumor stroma and CD163-expression by cancer cells. MI was categorized as no/low, moderate, and high. **a** Disease-free survival in relation to MI and independent of CD163-expression. **b** In BC patients having tumors with no/low MI, the expression of CD163 in tumor cells was significantly associated with shorter disease-free survival (*p* < 0.001). **c** Patients having tumors with moderate MI showed no difference in DFS in relation to CD163-expression by tumor cells. None of the patients who had CD163-positive tumors classified as high MI died due to breast cancer

No associations were found between MI and RFS or DFS. To investigate possible subgroups of clinical significance, combinations of CD163-expression in cancer cells and MI were investigated in relation to DFS. None of the patients who had CD163-positive tumors classified as high MI died due to BC. Interestingly, among patients with tumors classified as low MI, there was a significantly lower DFS for those patients with CD163-positive tumors compared to those with CD163-negative tumors (93 vs 273 months; *p* < 0.001; Fig. 3).

### In vitro study: plating efficiency and cell survival in relation to radiation

**Fig. 4:**
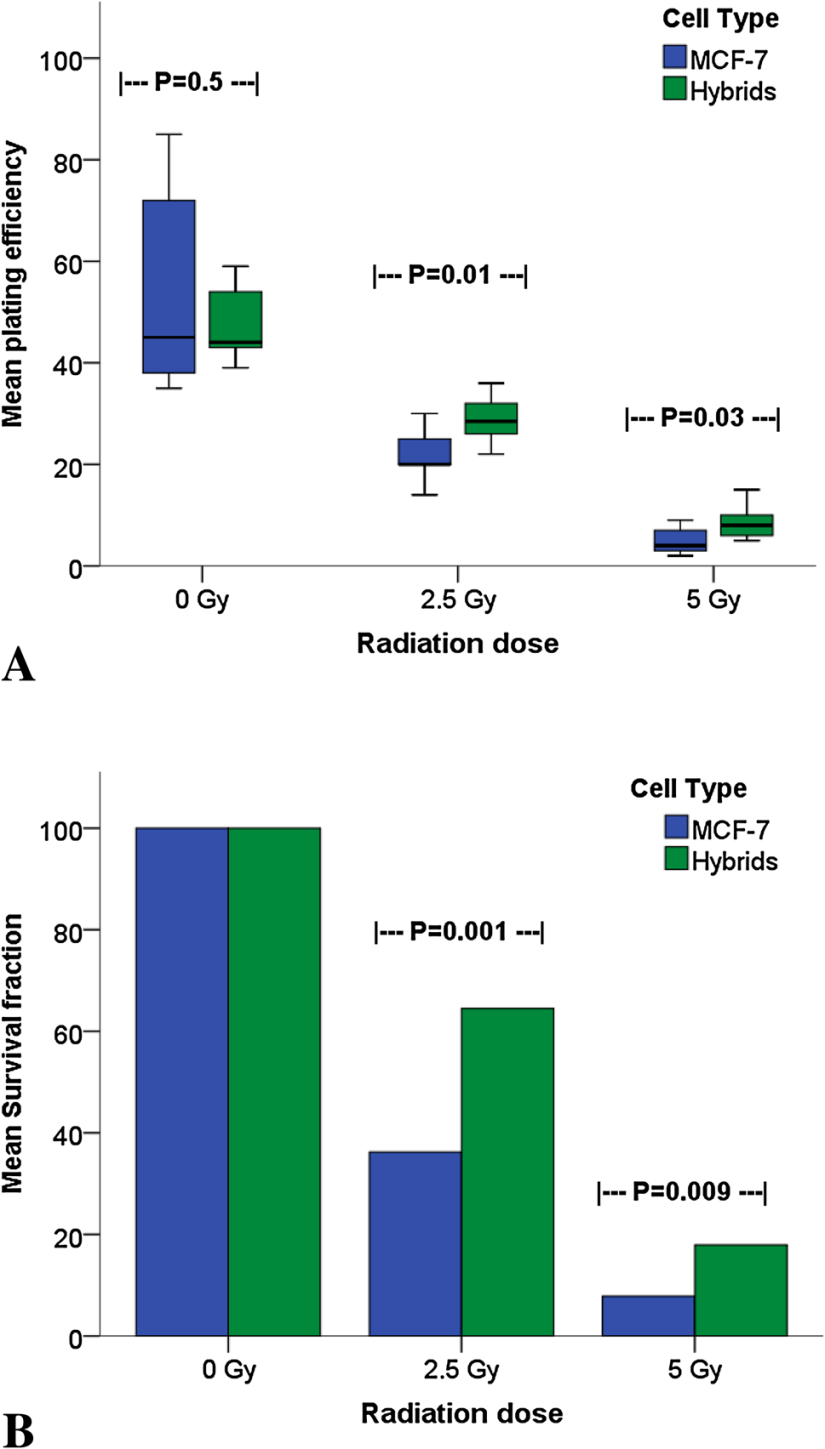
Plating efficiency (**a**) and survival fraction (**b**) of MCF-7 cells compared to macrophage:MCF-7 cell hybrids treated with 0–5 Gy γ-radiation. The 0 Gy value is considered as baseline value (control)

The generation rate of spontaneous hybrids (GFP-, CD163-, and CD45-positive) was estimated at an average of 2% (calculated in relation to the number of seeded macrophages). Using flow cytometry, these GFP-, CD163-, and CD45-positive cells were collected and their radiosensitivity investigated in relation to maternal MCF-7 cells. Colony forming ability, evaluated as plating efficiency (PE), was calculated after 0 Gy (control), 2.5, and 5 Gy γ-ionizing radiation. Both PE and SF decreased dose-proportionately in both hybrids and MCF-7 cells with no differences between MCF-7 and hybrid cells at 0 Gy. However, after both 2.5 and 5 Gy, hybrids had significantly higher PE than MCF-7 cells (*p* = 0.01 and *p* = 0.03 respectively, Fig. 4a). Similarly, the SF of the hybrids was nearly double that of MCF-7 cells following radiation of 2.5 Gy (65% as compared with 36%, *p* = 0.001) and surpassed double after 5 Gy (18% as compared with 8%, *p* = 0.009; Fig. 4b).

## Discussion

To our knowledge, this is the first study to investigate the macrophage phenotype of cancer cells in relation to radiotherapy response and survival in breast cancer. Our results suggest an association between CD163-expression in cancer cells and poor response to radiotherapy, as patients with CD163-positive tumors had significantly shorter DFS following postoperative radiotherapy as compared with those with CD163-negative tumors. Furthermore, our in vitro studies support our clinical observations in that macrophage:MCF-7-hybrids survived radiation and retained their colony-forming ability to a higher extent than their maternal MCF-7 cancer cells.

This study focuses on the interaction between tumor cells and immunological cells through two separate but seemingly interrelated perspectives: one, the number of TAMs (MI) and two, the macrophage traits as expressed by the tumor cells. Macrophages are known to infiltrate malignant tissues to a variable degree, eliciting either pro- or antitumor responses depending on the specific tissue microenvironment (Condeelis and Pollard 2006). TAMs influence tumor biology through paracrine interactions with cancer cells and may promote cancer cell proliferation and tumor progression (Biswas et al. 2008; Tsutsui et al. 2005). In breast cancer, high levels of MI have previously been associated with aggressive features, larger tumor size, increased proliferation index, and poor prognosis (Gwak et al. 2015; Medrek et al. 2012). The results of the current study are in agreement with these findings in that moderate and high MI were associated with increased Ki-67-expression and high grade (NHG 2–3).

Increased recruitment and infiltration of macrophages in tumor tissue are believed to increase the rate of fusion between macrophages and cancer cells. Cell fusion is a natural biological process in normal development and tissue regeneration and results in hybrid cells that express genetic and phenotypic properties from both maternal cells (Johansson et al. 2008). This phenomenon is a more efficient mechanism of DNA-exchange and cellular reprogramming than the accumulation of mutations in single cells (Bastida-Ruiz et al. 2016; Dittmar et al. 2013; Duelli and Lazebnik 2003). Growing in vitro (Busund et al. 2002, 2003; Shabo et al. 2015; Wei et al. 2014), in vivo (Powell et al. 2011; Silk et al. 2013), and clinical (LaBerge et al. 2017; Lazova et al. 2013; Yilmaz et al. 2005) data indicate that this process occurs in solid tumors and may play a significant role in clinical tumor progression. Moreover, this process generates malignant cell clones (hybrids) with reduced susceptibility to oncological treatments (Carloni et al. 2013; Nagler et al. 2011; Wang et al. 2012; Yang et al. 2010).

In vivo frequency of cell fusion is low, estimatedly up to 1% in experimental tumor models (Duelli and Lazebnik 2003), but the fusion efficiency increases proportionally to the malignancy of tumor cells (Miller et al. 1988) and presence of inflammation (Johansson et al. 2008). In a recent study, we showed that macrophage:MCF-7 hybrids can be generated spontaneously at an average rate of 2% (Shabo et al. 2015). One gram of tumor mass is assumed to contain approximately 1 × 10^8^ tumor cells (Del Monte 2009), suggesting theoretically that each gram of breast cancer tissue may potentially generate approximately 2 million hybrid cells. Thus, although the proportion of hybrids may be small in relation to the total number of malignant cells, the survival of the hybrids may generate a subset of therapy-resistant cells that might have important clinical implications.

Macrophage traits in breast cancer was first reported by our group in 2008 (Shabo et al. 2008). It was later reported in several other solid tumors, such as colorectal and bladder cancers (Aljabery et al. 2017; Maniecki et al. 2012; Shabo et al. 2009, 2014). Aljabery et al. reported that CD163-expression in bladder cancer cells was proportional to MI (Aljabery et al. 2017). Likewise, in the current study, the mean number of cancer cells expressing CD163 was positively associated with MI, supporting a logical connection between fusion events and the number of TAMs.

One interesting observation linked to MI and CD163-expression is our finding that among those classified as low MI, DFS was significantly shorter for patients whose tumors were CD163-positive as compared to CD163-negative. Although this result should be interpreted carefully due to low number of patients in our subgroup analysis, it may be pointed out that similar findings were observed by Aljabery et al. in bladder cancer (Aljabery et al. 2017). A protective effect of TAMs has been demonstrated both in vitro and in vivo (Ohkuri et al. 2017). Fidler (1988) showed that activation of macrophages eliminated cancer cells and reduced metastases, but this mechanism was limited by the ratio of macrophages in relation to target cancer cells (Fidler 1988). Thus, the clinical impact of macrophages is more complicated than simply determining their density in tumor stroma. The bidirectional influence of the phenotype of cancer cells and the immunological function of macrophages is likely to influence the clinical outcome of a tumor (Biswas and Mantovani 2010; Georgoudaki et al. 2016).

In conclusion, the findings presented in this study support the role of the macrophage phenotype in influencing radiological response in breast cancer. Further studies are warranted to investigate if this phenotype may be useful in identifying a subset of patients at greater risk for recurrence after radiotherapy and for future development of more efficacious treatments for this patient group.
